# Naloxone Metabolism Associated With Unexpected Noroxymorphone Detected in Urine During Pregnancy

**DOI:** 10.1097/og9.0000000000000188

**Published:** 2026-08-06

**Authors:** Marcela C. Smid, Jasmin E. Charles, Gwendolyn A. McMillin

**Affiliations:** Department of Obstetrics and Gynecology, Division of Maternal Fetal Medicine, and Department of Pathology, University of Utah Health, Salt Lake City, Utah; and NMS Laboratories, Horsham, Pennsylvania.

## Abstract

**BACKGROUND:**

Urine drug testing (UDT) is commonly used in obstetrics to monitor opioid use disorder treatment, but interpretation can be difficult and may trigger harmful consequences, including child welfare reporting. Noroxymorphone, also referred to as nornaloxone, may arise from metabolism of naloxone, oxycodone, or oxymorphone and may be detected in patients taking buprenorphine/naloxone.

**CASES:**

Two pregnant individuals receiving buprenorphine/naloxone had unexpected presumptive positive opiate screens. Confirmatory testing showed isolated low-level noroxymorphone (46 and 79 ng/mL) without detectable parent opioids, consistent with naloxone metabolism rather than opioid use. Both patients experienced significant distress after the initial results.

**CONCLUSION:**

Clinicians should recognize naloxone-associated noroxymorphone to avoid misclassifying return to use. Confirmatory testing and interpretation are essential to prevent harm and preserve clinician–patient trust.

## Abstract 2

In pregnant patients receiving buprenorphine/naloxone, isolated noroxymorphone detection may reflect naloxone metabolism, risking misclassification of return to opioid use.

**Teaching Points**

- Isolated noroxymorphone test results among patients receiving buprenorphine/naloxone may reflect naloxone metabolism rather than opioid return to use.
- Unexpected or clinically discordant urine drug testing results during pregnancy should undergo confirmatory mass spectrometry before changes in treatment or child welfare reporting are considered.
- Counseling patients about the purpose, limitations, and interpretation of perinatal urine drug testing may reduce anxiety, strengthen therapeutic trust, and support ongoing engagement in addiction treatment.

Urine drug testing (UDT) is frequently used in obstetric clinics and labor and delivery units to monitor engagement in medication for opioid use disorder, to identify ongoing substance exposure, and to guide peripartum clinical decision making. However, routine perinatal toxicology testing presents serious challenges, including limited clinician training in result interpretation and substantial potential for psychosocial and legal harm when results are misunderstood or applied punitively.^1–5^ In a large cohort evaluating delivery-based UDT, unexpected positive results were uncommon yet frequently resulted in child welfare referral, underscoring how even rare or ambiguous findings may have consequences when interpreted without sufficient knowledge and clinical contextualization.^2^

Within this landscape, clinicians also demonstrate significant knowledge gaps. A survey of obstetric and pediatric clinicians found only fair proficiency in toxicology interpretation.^4^ In the obstetric context, routine UDT without accurate interpretation may amplify stigma and contribute to inequitable child welfare involvement.^6^ Accurate interpretation of nuanced laboratory findings is critical. Noroxymorphone detection represents one such interpretive challenge that can directly affect patient trust, continuity of care, and potential legal outcomes.

We present two cases of noroxymorphone-positive urine toxicology results that caused substantial anxiety to pregnant patients taking buprenorphine/naloxone as prescribed. Both cases are presented at the time of the discrepant UDT. Written informed consent for publication was obtained from both individuals. To contextualize these findings, we include previously unpublished laboratory data demonstrating that naloxone metabolites may trigger opiate immunoassay positivity.

## CASES

### Case 1

A 38-year-old gravida 6 para 3-0-2-3 patient at 30 4/7 weeks of gestation with opioid use disorder in sustained remission (with all UDT consistent with only prescribed medication use during pregnancy) was followed up in an integrated perinatal addiction clinic and prescribed buprenorphine/naloxone. Her medical history included resolved hepatitis C infection, remote injection drug use complicated by internal jugular thrombosis, recurrent urinary tract infections, and advanced maternal age. She demonstrated stable engagement in prenatal care with no clinical concern for opioid return to use. Routine UDT results included a positive point-of-care opiate immunoassay with buprenorphine detected. Confirmatory liquid chromatography–tandem mass spectrometry testing identified noroxymorphone at 46 ng/mL with no parent opioids, including oxycodone, oxymorphone, hydrocodone, hydromorphone, morphine, codeine, 6-monoacetylmorphine, noroxycodone, and norhydrocodone. Buprenorphine and norbuprenorphine metabolites were identified, consistent with prescribed medications.

After consultation with laboratory specialists, these findings were interpreted as consistent with naloxone metabolism rather than opioid return to use. No changes were made to her treatment regimen. The patient reported significant emotional distress after receipt of the screening result. Detailed explanation of results supported continued clinical stability. Subsequent UDT results were consistent with prescribed medications.

### Case 2

A 25-year-old gravida 5 para 0-0-4-0 patient at 39 0/7 weeks of gestation was similarly followed up in an integrated perinatal addiction program and prescribed buprenorphine/naloxone. Psychiatric comorbidities included generalized anxiety disorder, borderline personality disorder, posttraumatic stress disorder, depression, and nicotine use disorder. She reported extensive lifetime trauma, including physical and sexual abuse and past intimate partner violence. She was recently discharged from residential treatment and was residing in supportive housing while awaiting sober living placement. She also demonstrated stable prenatal engagement and UDT results that were consistent with prescribed medication throughout her prenatal care, with no clinical concern for opioid return to use.

Labor and delivery admission screening UDT demonstrated a presumptive positive opiate immunoassay. She reported recent poppy seed ingestion immediately before labor and delivery admission. Confirmatory liquid chromatography–tandem mass spectrometry testing detected noroxymorphone at 79 ng/mL and no detectable morphine, codeine, oxycodone, oxymorphone, hydrocodone, hydromorphone, or fentanyl or their metabolites. A repeat urine screen 3 days later was negative. In consultation with a perinatal addiction specialist, results were interpreted as naloxone metabolism rather than opioid return to use. During delivery hospitalization, the patient experienced significant anxiety that the result might trigger child welfare reporting despite adherence to treatment. Multidisciplinary consultation and explanation of confirmatory results were required to provide reassurance to the patient and staff and to avoid a child welfare referral. Subsequent UDT results for this patient was consistent with prescribed medications.

## DISCUSSION

These two cases demonstrate that isolated detection of noroxymorphone during pregnancy may reflect naloxone metabolism rather than opioid return to use, a UDT interpretation issue not previously addressed in the perinatal addiction literature. Although publications have highlighted the ethical, legal, and psychosocial challenges of perinatal drug testing, including stigmatization, punitive responses, and care disruption,^3^ they have not addressed metabolite-level interpretation for patients receiving buprenorphine/naloxone.

**Fig. 1.**
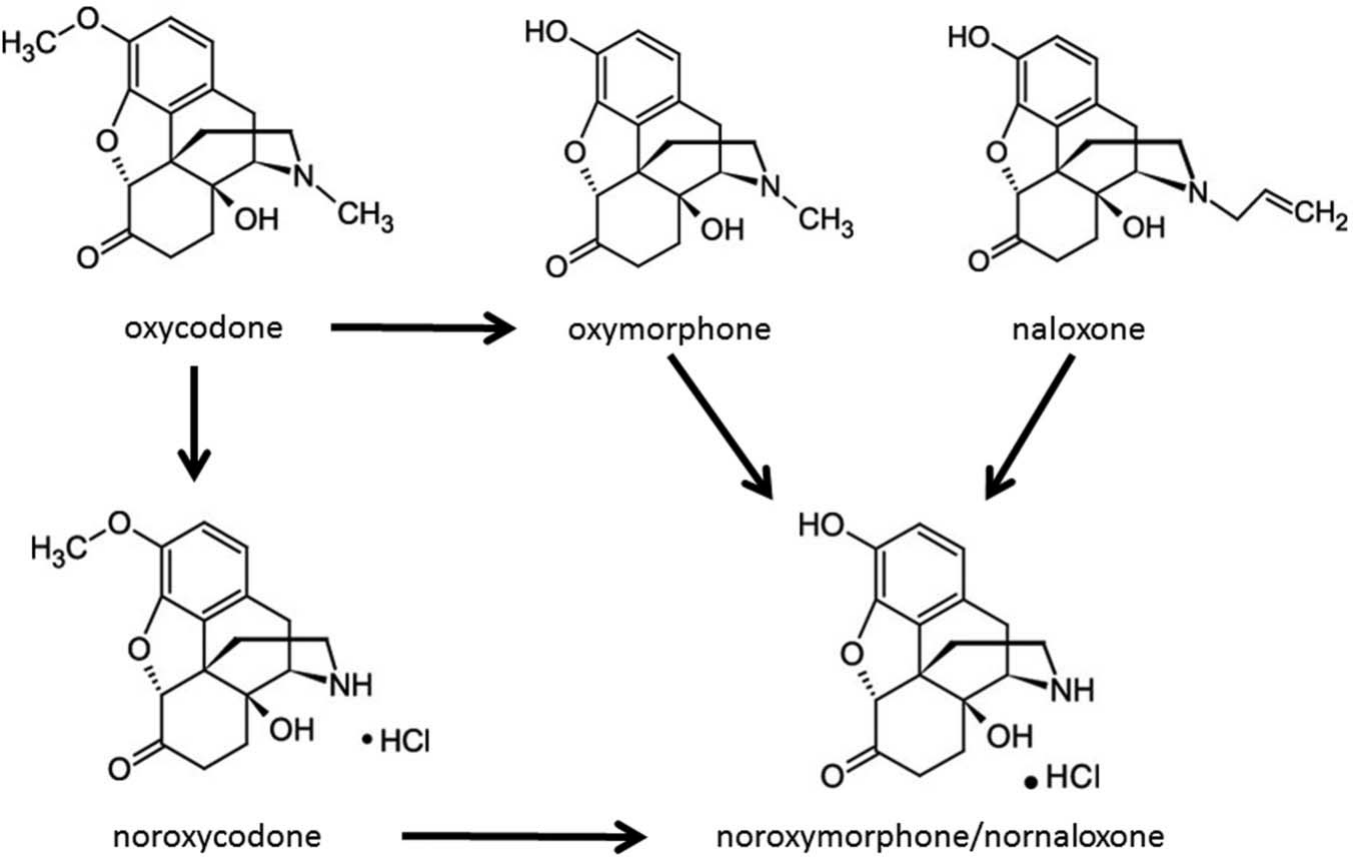
Metabolic pathways leading to noroxymorphone: contributions of oxycodone, oxymorphone, and naloxone.

Outside of obstetrics, toxicology literature has clarified that noroxymorphone (nornaloxone) may derive from metabolism of oxycodone, oxymorphone, or naloxone (Fig. 1).^7,8^ In response to these findings, ARUP Laboratories performed a retrospective review of 14,587 deidentified urine specimens that tested positive for noroxymorphone and for oxycodone, oxymorphone, or noroxycodone (cutoff 20 ng/mL) by liquid chromatography–tandem mass spectrometry. Median noroxymorphone concentration was 183 ng/mL, and the 25th percentile of the dataset was 75 ng/mL. To identify samples with noroxymorphone derived from naloxone, 168 urine specimens negative for oxycodone, oxymorphone, and noroxycodone and positive for buprenorphine or metabolites were prospectively acquired and evaluated. Of these 168 specimens, median noroxymorphone concentration was 36 ng/mL, mean was 44 ng/mL, SD was 39 ng/mL, and maximum was 296 ng/mL. Among these specimens, 26 (15.5%) had noroxymorphone concentrations of 75 ng/mL or more. Because these were deidentified samples, buprenorphine/naloxone dosing was not known, and results were not normalized to creatinine. Although some overlap in concentrations is expected, these data demonstrated higher noroxymorphone concentrations when derived from oxycodone or oxymorphone than when associated with naloxone exposure in most patients. Of note, noroxymorphone is also a minor metabolite of naltrexone, suggesting that unexpected detection of noroxymorphone may result from naltrexone use.

*Smid. Naloxone Metabolism in Pregnancy. O&G Open 2026*.

In our cases presented here, oxycodone, noroxycodone, and oxymorphone results were negative by liquid chromatography–tandem mass spectrometry, and noroxymorphone concentrations (46 and 79 ng/mL) were closer to the median associated with naloxone metabolism (36 ng/mL) than to that associated with oxycodone or oxymorphone metabolism (183 ng/mL). These results support therapeutic naloxone exposure as the most likely source rather than unexpected opioid use. Noteworthy, noroxycodone is not expected if a person is using only naloxone because noroxycodone is not an expected metabolite, precursor, or contaminant. Noroxymorphone, however, would be an expected metabolite when only naloxone or potentially naltrexone is used if UDT is performed within approximately 24 hours after use of naloxone. Approximately half of a naloxone dose is eliminated within the first 6 hours after administration.^9^

These interpretive challenges arise within a broader context of structural inequities in perinatal toxicology testing. In a retrospective review of more than 2,000 peripartum dyads, only 3.9% had unexpected toxicology findings, yet nearly half of these cases led to child protective services referral despite limited clinical documentation of substance use.^10^ The authors concluded that the low yield of unexpected positives and disproportionate social consequences warrant caution regarding routine toxicology testing and its use as a trigger for punitive action. Multiple studies demonstrate that Black pregnant patients and those with publicly insured or low-income status are disproportionately subjected to screening despite similar positivity rates compared with White patients.^10–13^ Compounding the disparities in who is tested, studies of labor and delivery units and child welfare reporting show that a positive UDT at delivery is strongly associated with child welfare referral and that these referrals disproportionately affect Black and publicly insured patients, even when the clinical benefits of testing are uncertain.^5,14^ Professional organizations, including the American College of Obstetricians and Gynecologists and the Society for Maternal-Fetal Medicine, caution that such practices may contribute to stigma, child welfare involvement, avoidance of prenatal care, and deterioration of patient–clinician trust.^15,16^

At our institution, UDT is obtained with explicit patient consent, and routine testing on labor and delivery for patients with known substance use disorder is implemented through multidisciplinary collaboration among obstetric, pediatric, and social work teams. Although designed to support maternal–infant safety and care coordination, these cases illustrate that even consent-based, protocolized testing remains limited by interpretive complexity and potential for unintended harm. Ongoing multidisciplinary discussions are focused on refining best practices to balance clinical utility with trauma-informed, equity-centered care and to minimize unnecessary child welfare involvement. Consideration of a toxicology interpretation service, similar to cardiology interpretation of electrocardiograms, has been proposed to provide consultation in the setting of complex or discrepant results. In the absence of a toxicology service, clinicians can consult with laboratory directors where screening or testing was performed or seek guidance from medical toxicologists. Laboratories frequently have unexpected results algorithms to guide clinicians with suggestions for testing procedures.^17,18^ In both cases, screening results produced substantial patient distress, including explicit fears of child welfare services involvement despite stable treatment engagement. Counseling about testing purpose and discrepant results may reduce patient anxiety. These experiences emphasize that UDT interpretation during pregnancy not only is a technical matter but also shapes psychologic safety, therapeutic alliance, and continuity of perinatal addiction treatment.

Effective care for pregnant patients receiving buprenorphine/naloxone therefore requires confirmatory mass spectrometry for unexpected or clinically discordant results, application of emerging concentration-based thresholds (recognizing that isolated noroxymorphone less than 75 ng/mL without parent opioids likely reflects naloxone metabolism), and reconciliation of prescribed medications and potential dietary exposures before escalation or reporting decisions. Accurate metabolite interpretation is essential to providing safe, equitable, and patient-centered perinatal addiction care.
